# The Prevalence and Effect of Comorbid Cystic Fibrosis and Attention Deficit Hyperactivity Disorders on Hospitalizations: A Retrospective Analysis

**DOI:** 10.7759/cureus.3048

**Published:** 2018-07-25

**Authors:** Nicole Spitzer, Timothy B Legare, Priyanshi Patel, Nicholas Toselli, Floyd Livingston

**Affiliations:** 1 Ophthalmology, University of Central Florida College of Medicine, Orlando, USA; 2 Surgery, University of Central Florida College of Medicine, Orlando, USA; 3 Pediatrics, University of Central Florida College of Medicine, Orlando, USA; 4 Miscellaneous, University of Central Florida College of Medicine, Orlando, USA; 5 Pediatric Pulmonology, Nemours Children's Hospital, Orlando, USA

**Keywords:** cystic fibrosis, attention deficit hyperactivity disorder, treatment compliance, adhd, adherence, adherence

## Abstract

Introduction: The prevalence of attention-deficit/hyperactivity disorder (ADHD) in pediatric cystic fibrosis (CF) patients is comparable to the general population, but the effects of ADHD on CF treatment and the outcomes have been minimally investigated.

Methods: Two cohorts were retrospectively reviewed, pediatric patients with comorbid CF/ADHD and patients with CF only. Each patient with CF/ADHD was age and sex-matched to a CF-only patient based on their most recent pulmonary office visit. Each patient was reviewed for forced expiratory volume in one-second percent predicted (FEV1%pred), body mass index (BMI) percentile, and hospitalizations for one year prior to the last pulmonary visit.

Results: A total of 624 patients with CF were identified, with 52 having co-morbid CF/ADHD (8.3%). Of those identified, 46 met inclusion criteria and were analyzed in the CF/ADHD cohort. The mean total hospital admissions between the CF/ADHD cohort and the CF-only cohort were not statistically significant (2.22 vs 1.834, p=.467). The difference between the BMI percentiles was not statistically significant (48.634 vs 38.634, p=.135). The difference between FEV1%pred was statistically significant at 84% for the CF/ADHD group and 74% for the CF-only group (p=.042).

Conclusion: The difference in total hospital admissions between the CF/ADHD cohort and the CF-only cohort did not reach statistical significance, but the study was underpowered. There was a significant difference between FEV1%pred between the two groups, in favor of the comorbid CF/ADHD population. More research is needed to further evaluate the effects of a comorbid ADHD diagnosis on outcomes in the CF pediatric population.

## Introduction

Cystic fibrosis (CF) is an autosomal recessive disorder that is caused by a mutation in the CF transmembrane conductance regulator (CFTR) protein [1]. The CFTR protein is a cycloadenosine monophosphate regulated chloride channel that regulates sodium and water transport in and out of epithelial cells [1]. A defect in this channel leads to thick secretions in many locations in the body such as the respiratory system, gastrointestinal tract, sweat glands, and various other places [1,2]. CF is the most common autosomal recessive life-shortening disorder amongst Caucasians [3]. Patients experience recurrent bronchopulmonary infections, malabsorption, pancreatic insufficiency, and nutritional derangements [1]. CF has been described as a life-shortening disorder, however, recent changes in disease management over the past 20 years have increased life expectancy from an average age of 28 to 37 [1,4]. Improvement in life expectancy is due to the increased availability and treatment of the manifestations of CF, as there currently is no cure.

The multisystem effect of CF leads to treatments being complex, and treatments may utilize a variety of different strategies: airway clearance, inhalants to alter airway surface liquids and mucus, antimicrobials for infections, anti-inflammatory medications, medications that target the defective CFTR protein, and nutritional therapy [1]. A review of the literature in 2014 showed that the average number of self-administered home treatments for a CF patient is seven, taking an average of 108 minutes per day to perform [4]. Due to this complexity, treatment adherence has varied widely amongst pediatric CF patients [4]. Treatment adherence has been shown to decrease morbidity, mortality, and hospitalizations [4-5]. As CF patients grow older and the prognosis of CF improves, it becomes imperative for physicians to determine the barriers to adherence and the risk factors for non-adherence and determine how best to intervene in non-adherent patients.

Another area currently being investigated is the effect of chronic illnesses on developing psychiatric illnesses. In general, patients with chronic diseases are at a greater risk for psychiatric, emotional, and behavioral disorders, given the complexity surrounding therapy and the burden of the disease [6-7]. Pediatric CF patients appear to have a significant risk of developing psychological manifestations, such as depression and anxiety [8-9]. Patients with CF associated with other co-morbidities, such as depression, have an increased hospitalization risk [10].

Attention-deficit/hyperactivity disorder (ADHD) is the most common behavioral disorder found in the pediatric population and should be considered as a diagnosis for any child older than four years old who presents with distractibility, hyperactivity, impulsiveness, poor academic performance, behavioral problems, or poor attention spans [11]. The ADHD diagnosis is based upon the Diagnostic and Statistical Manual of Mental Disorders (DSM-V) and is further defined by three subtypes: primarily inattentive, primarily hyperactive, and combined subtypes [11]. Most recent data suggests the prevalence of ADHD is increasing, from 7.8% in 2003 to 9.5% in 2007 [11-13]. According to the Centers for Disease Control and Prevention, as of 2011, approximately 11% of children four to 17 years of age have been diagnosed with ADHD in the United States [14].

The current data on comorbid ADHD and CF is sparse. To date, our team identified three articles that address ADHD in the pediatric CF population. The first, published in 2011, found that the prevalence of ADHD diagnoses in the CF population at Massachusetts General Hospital (9.6%) mirrored the prevalence in the general pediatric population (10%) [15]. The second study reported the prevalence of ADHD diagnoses in the CF population of Medicaid patients from 1999-2006 [16]. This study reported an increase in the prevalence of CF/ADHD patients from 5.26% in 1999 to 8.16% in 2006 [16]. Finally, the most recent study, in 2018, found that in their pediatric and adult CF population, there was a higher rate (17%) of ADHD symptoms than in the general population [17]. Diagnosing and managing ADHD in CF patients may become paramount because of the increased complexity of treatment for CF and the impact that treatment adherence has on outcomes in this population. It has been found that ADHD is associated with impairment of self-care, and data has suggested that the impulsivity of ADHD may be associated with early mortality [15,18]. Most children with ADHD present with poor attention spans, task avoidance, distractibility, deficits in impulse control and executive function, and other behavioral problems [12,15]. Deficiencies in executive functioning can present as forgetfulness and difficulty in planning and coordinating everyday tasks [19]. This may result in trouble following complex directions and may create a risk for errors in managing difficult treatment plans [11-12,20]. These associations warrant investigations into the effects of comorbid CF/ADHD and its impacts on healthcare outcomes. In this study, we will report the prevalence of ADHD in our pediatric CF population at a multisite tertiary care pediatric hospital system. We hypothesize that there will be significant differences in the number of hospitalizations, BMI percentiles, and pulmonary function tests between patients with comorbid ADHD/CF and patients with CF only.

## Materials and methods

Methods

Following institutional review board approval by the Nemours Office of Human Subjects Protection (741996), a retrospective cohort study of all patients diagnosed with CF and comorbid CF/ADHD from 1/1/04 to 12/31/14 was conducted. As a first step, the extraction of patients with the ICD-9 code for CF was performed. Additionally, the medical records extracted (n = 624) were queried for the presence of the ICD-9 code for ADHD (n=52). Data were extracted from medical records from all four children's hospitals in our institution. CF and CF/ADHD patients were matched by age, sex, and most recent clinic visit (within ±6 months of matched cohort) to a patient who had CF without an ADHD diagnosis (CF-only cohort). Of the 52 comorbid CF/ADHD patients, one patient was unable to be matched to a CF only patient, two had incomplete spirometry records for comparison, and three patients were determined from the chart review to not have ADHD. The incomplete records were consistently seen in patients who either transferred care or only had one office visit.

Inclusion criteria for the CF/ADHD arm of the study were as stated: age greater than six years old, diagnosis of cystic fibrosis with pulmonary manifestations, and a diagnosis of any subtype of ADHD. Inclusion criteria for the CF-only arm were as stated above, without a diagnosis of any subtype of ADHD.

Variables

For each patient that met the inclusion criteria, the following variables were extracted: the CF/ADHD cohort was reviewed at their most recent clinic date for age, sex, race, BMI percentile, and forced expiratory volume at one-second percent predicted (FEV1%pred). Additional variables included: number of hospitalizations within one year, along with whether the patient had visited the emergency room (ER) with no admission to the hospital, was seen in the emergency room, and had been admitted to the hospital (ER-ADM), and whether the patient had been admitted to the hospital without visiting the emergency room (ADM). Each patient was then grouped into categories based upon severity using their FEV1%pred at the most recent office visit, with categories defined as: Mild CF = FEV1%pred >70%, Moderate CF = FEV1%pred <70%, but >40%, and Severe CF = FEV1%pred <40%.

Data management and statistical analyses

Sex and ethnicity are reported as frequency and percentage. BMI, FEV1%pred, and number of hospitalizations are reported as means with standard deviation. An independent t-test was used to assess group differences for hospitalization data. A Pearson Chi-square test (X2) was used to assess the associations of sex, FEV1%pred, and BMI. The alpha level for significance was set at .05. All statistical analysis were two-tailed and conducted in SPSS (IBM Corp. Released 2015. IBM SPSS Statistics for Windows, Version 23.0. Armonk, NY, IBM Corp.).

## Results

There were a total of 624 patients identified with CF at our institution. A total of 52 patients (8.3%) were identified to have comorbid CF/ADHD. Of the 572 CF-only patients, 338 (54.2%) were male and 286 (45.8%) were female. Of the 52 patients with comorbid CF and ADHD, 39 (75%) were male and 13 (25%) were female. This is statistically significantly different from the male to female distribution in the CF-only cohort (52.3% male vs. 45.8% female, p=.002).

CF/ADHD arm

A total of 46 patients with CF/ADHD were identified and met the inclusion criteria. There were 36 males with CF/ADHD and 10 females with a mean age of 14.7 years (range 6-21 years) at the initial office visit. The mean BMI percentile was 48.634% (SD= 31.736). Amongst the CF/ADHD group, there were 39 (84.8%) Caucasians, three (6.5%) African-Americans, three (6.5%) Hispanic patients, and 1 (2.2%) designated as “other; race not specified.” A compilation of demographic statistics is shown in Table 1. The average FEV1%pred of the CF/ADHD group was 84.70% (SD=21.637). Of the 46 patients in the CF/ADHD arm, 35 (76.1%) fell into the Mild CF category, 10 (21.7%) in the Moderate CF, and 1 (2.2%) with Severe CF. Pulmonary function and BMI percentile comparisons are illustrated in Table 2.

**Table 1:** Matched Patient Demographics CF: Cystic Fibrosis; ADHD: Attention-Deficit/ Hyperactivity Disorder

	CF/ADHD	CF Only
Total Patients (n=92)	46	46
Female	10 (21.7%)	10 (21.7%)
Male	36 (78.3%)	36 (78.3%)
African American	3 (6.5%)	4 (8.7%)
Caucasian	39 (84.8%)	39 (84.8%)
Hispanic	3 (6.5%)	2 (4.3%)
Other (not specified)	1 (2.2%)	1 (2.2%)
Mean age (range)	14.7 years (6-21)	14.8 years (6-21)

**Table 2:** BMI Percentile, Pulmonary Function, and Hospital Admission Statistics BMI: Body Mass Index percentile; FEV1%pred: forced expiratory volume in one second percentile; ADM: Direct hospital admission; ER-ADM: Emergency room to hospital admission; ER: Emergency room visit with no hospital admission

	ADHD/CF	CF Only
Mean BMI percentile (%)	48.634% (SD=31.736)	38.643% (SD=28.536)
Mean FEV1%pred	84.70% (SD= 21.637)	74.76% (SD=24.377)
Mild CF (FEV1%pred>70%)	35 (76.1%)	32 (69.6%)
Moderate CF (FEV1%pred =40-70%)	10 (21.7%)	9 (19.6%)
Severe CF (FEV1%pred <40%)	1 (2.2%)	5 (10.9%)
Average Total Hospital Admissions	2.22 (n=18, SD 2.340)	1.826 (n=23, SD .984)
ADM	16 (34.6%)	19 (41.3%)
ER-ADM	4 (8.7%)	7 (15.2%)
ER	4 (8.7%)	1 (2.2%)
No Hospital Visit	22 (47.8%)	19 (41.3%)

CF arm

A total of 46 patients were analyzed that met inclusion criteria: There were 36 males and 10 females with an average age at the time of analysis of 14.8 years (range 6-21 years) and a mean BMI percentile of 38.643% (SD=28.536). Amongst the CF-only group, there were 39 (84.8%) Caucasians, four (8.7%) African-Americans, two (4.3%) Hispanic patients, and one (2.2%) patient designated as “other; race not specified”; see also Table 1. The mean FEV1%pred for the CF only arm was 74.76% (SD=24.377). Of the 46 patients in the CF-only arm, 32 (69.6%) fell into the Mild CF category, 9 (19.6%) in the Moderate CF category, and 5 (10.9%) into the Severe CF category; see also Table 2.

CF/ADHD to CF comparisons

Since the groups were matched based on age and sex, the differences were non-significant (p=.995 and p=1.0, respectively). The race and BMI percentile, which were not matched variables, were also not statistically significant between the two groups (p=.952 and p=.135, respectively). With regards to hospital admissions, only the most recent pulmonary visit was a statistically significant difference in the mean FEV1%pred between CF/ADHD (mean=84.70% (SD=21.637) predicted) patients and CF-only (mean=74.76% (SD=24.377) predicted, p=.042) patients. The mean total admissions for the CF/ADHD arm was 2.22 (SD=2.34) and for the CF-only group was 1.826 (SD=.984). Other hospital admission data is represented in Table 2.

## Discussion

The prevalence of ADHD in the pediatric CF population at our institution was 8.3%, which is comparable to that of the general population per previous data that suggest a range of prevalence from 7.8%-11% [13-15]. Without being able to see immediate results, managing an increasingly more difficult and time-intensive treatment regimen every day for life becomes a burden for adolescent patients with CF [21]. On average, CF patients exhibit poor treatment adherence and this is correlated with a decrease in lung function and an increase in lung exacerbations [22]. In patients with comorbid ADHD and CF, the underlying changes in executive functioning and behavior can impair self-care skills and disrupt treatment adherence. A key motivating factor in the treatment adherence of CF patients is the early development of self-care skills through repeated practice and encouragement [21]. ADHD can interfere with the development of self-care skills, potentially removing an integral treatment motivator and possibly leading to worse outcomes in CF patients. An ADHD diagnosis in the CF population may not be recognized immediately by caregivers because they may attribute the behavioral changes to symptoms of the disease or complications from treatments of the chronic illness that interfere with the patient’s activities of daily living [15]. The early identification and treatment of ADHD in CF patients may improve healthcare management and increase treatment adherence. There is a need for a better understanding of the impact that traditional ADHD treatment has on the progression of CF, including treatment adherence and changes in life expectancy and/or quality of life. Evidence shows that drug therapies, such as stimulants, non-stimulants, and combination therapies, are good treatment options for ADHD, but the literature is lacking on how each of these treatments would affect CF patients with differing disease severities [15].

While the primary outcome of total hospital admissions was not statistically significant between the CF/ADHD group and the CF-only group, the data yielded results that warrant further investigation. The CF/ADHD group had statistically significantly better FEV1%pred, which is generally considered to be a clinical indicator of good pulmonary health in CF but had more non-statistically significant frequent hospitalizations [23-25]. This is paradoxical because treatment adherence in CF is already known to be difficult and ADHD has been thought to increase the difficulty [4,26-27]. Additionally, a recent study has shown that increasing treatment adherence reduces pulmonary exacerbations and may lead to fewer hospitalizations [22]. Since the treatment of CF is complex and time-consuming, ranging from 108 minutes a day to two hours per day, and because patients do not see immediate benefits, treatment adherence is a major concern in the CF population [4,21]. Many factors ultimately determine treatment adherence, including socioeconomics, clinician-patient relationship, education about the consequences and sequelae of non-adherence, development of self-care skills, and others [28-29]. ADHD may be increasing or decreasing the adherence in CF patients, leading to the increased hospitalizations and increased FEV1%pred. The answer is not known at this time and, ultimately, the answer to this difference in hospitalizations may be multifactorial and complex.

Admittedly, there are some limitations to our work: we did not investigate whether or not the patients with comorbid CF/ADHD were being treated for their ADHD symptoms, but the pharmacotherapy for ADHD can result in weight loss and loss of appetite, which is opposite of the BMI percentile data we found [30], thus further inquiry into these findings is needed. Although our institution is a multi-site, not for profit children’s specialty care hospital operating in two states, we only identified 46 patients with CF/ADHD and complete records; with more data, we could address the issue of sample size, which has affected our study power.

## Conclusions

As more children with CF are living longer, it has become imperative to identify and treat comorbidities. Our work contributes to the limited research done on the prevalence of ADHD and CF, which shows that ADHD is diagnosed in the same percentage of the pediatric CF population as in the general pediatric population. There were differences in lung function, hospitalizations, and BMI between the two groups, although not statistically significant. Subsequent studies are needed to further elucidate the effect of a CF/ADHD co-morbid diagnosis on CF outcomes.
